# Supplementary material for: Angiotensin II induces coordinated calcium bursts in aldosterone-producing adrenal rosettes
Source: Nat Commun. 2020 Apr 3;11:1679. doi: 10.1038/s41467-020-15408-4 (PMC7125102; doi:10.1038/s41467-020-15408-4)
Supplement: Supplementary file 1 — Supplementary Information [file 41467_2020_15408_MOESM1_ESM.pdf]

Supplementary Information for

Coordinated Calcium Burst Firing within the Aldosterone-Producing Adrenal Rosette.

Nick A. Guagliardo, Peter M. Klein, Christina A. Gancayco, Adam Lu, Sining Leng, Rany R. Markem, Chelsea Cho, Craig G. Rusin, David T. Breault, Paula Q. Barrett and Mark P. Beenhakker

Corresponding author: Mark Beenhakker
Email: mpb5y@virginia.edu

This PDF file includes:

Figs. S1 to S6
Caption for movie S1

Other supplementary materials for this manuscript include the following:

Movie S1

Figure S1

a.

Gaussian-Exponential Intersection

$$y = \lambda \frac{1}{\sigma\sqrt{2\pi}} e^{\frac{-(x-\mu_1)^2}{2\sigma^2}} + (1-\lambda)\frac{1}{\mu_2} e^{\frac{-x}{\mu_2}}$$

	λ	μ1	σ	μ2
**50pM**	0.8555	2.034	0.7142	32.6169
**300pM**	0.8157	1.9884	0.7334	16.5089
**3nM**	0.7739	2.2944	0.8121	8.1901
**1uM**	0.8132	1.9924	0.6444	6.7698

b.

Bodova

$$y = (1-pSQ)\frac{1}{\sigma_1\sqrt{2\pi}} e^{\frac{-(x-\mu_1)^2}{2\sigma_1^2}} + pSQ * pQS \frac{1}{\sqrt{\sigma_1^2+\sigma_2^2}\sqrt{2\pi}} e^{\frac{-(x-(\mu_1+\mu_2))^2}{2(\sigma_1^2+\sigma_2^2)}} + \cdots$$

$$pSQ * (1-pQS) * pQS \frac{1}{\sqrt{\sigma_1^2+2\sigma_2^2}\sqrt{2\pi}} e^{\frac{-(x-(\mu_1+2\mu_2))^2}{2(\sigma_1^2+2\sigma_2^2)}} + \cdots$$

$$pSQ * (1-pQS)^2 * pQS \frac{1}{\sqrt{\sigma_1^2+3\sigma_2^2}\sqrt{2\pi}} e^{\frac{-(x-(\mu_1+3\mu_2))^2}{2(\sigma_1^2+3\sigma_2^2)}} + \cdots$$

$$pSQ * (1-pQS)^3 * pQS \frac{1}{\sqrt{\sigma_1^2+4\sigma_2^2}\sqrt{2\pi}} e^{\frac{-(x-(\mu_1+4\mu_2))^2}{2(\sigma_1^2+4\sigma_2^2)}}$$

	**pSQ**	**pQS**	μ1	σ1	μ2	σ2
**50pM**	0.1161	0.8967	2.0995	0.7942	2.2367	60.7067
**300pM**	0.1313	0.7637	2.0734	0.8410	2.8692	23.3050
**3nM**	0.1211	0.8764	2.3865	0.9941	2.6421	13.0360
**1uM**	0.1273	0.9411	2.0196	0.6797	1.5021	7.5822

c.

Thresholds

	**Gaussian + Exponential**	**Bodova**
**50pM**	4.2479	4.3361
**300pM**	4.0438	4.9425
**3nM**	4.3334	5.0286
**1uM**	3.6847	3.5216

Supplementary Figure 1. Calculations for determining a threshold value for defining inter-burst vs. intra-burst spikes. (a) Fitted parameters for a Gaussian-Exponential distribution fit to the inter-calcium spike interval distribution. This model assumes that bursts occur as a Poisson process (with inter-arrival times drawn from an exponential distribution with mean μ_2) and that inter-spike intervals within bursts are drawn from a Gaussian distribution $N(\mu_1, \sigma_1)$. The two distributions are combined with a weight λ between 0 and 1. **(b)** Fitted parameters for a multi-Gaussian distribution fit to the inter-calcium spike interval distribution based on the sub-threshold oscillation model proposed by Bodova et al. This model assumes that a cell is in either a quiescent (Q) state or a spiking (S) state. The state transitions occur as a Markov process with transition probabilities pSQ and pQS and with the inter-arrival times for states S and Q drawn from a Gaussian distributions $N(\mu_1, \sigma_1)$ and $N(\mu_2, \sigma_2)$ respectively. **(c)** Thresholds (seconds) for the maximum intra-burst interval as calculated from the Gaussian-Exponential model or the multi-Gaussian model (Bodova) for each dose of Ang II. For the Gaussian-Exponential model, the intersection between the Gaussian and exponential component is used. For the Bodova model, the sum of the mean quiescent (Q) state period and the mean spiking (S) state period is used, as the minimum sequence for burst separation is SQS under this model.

Figure S2

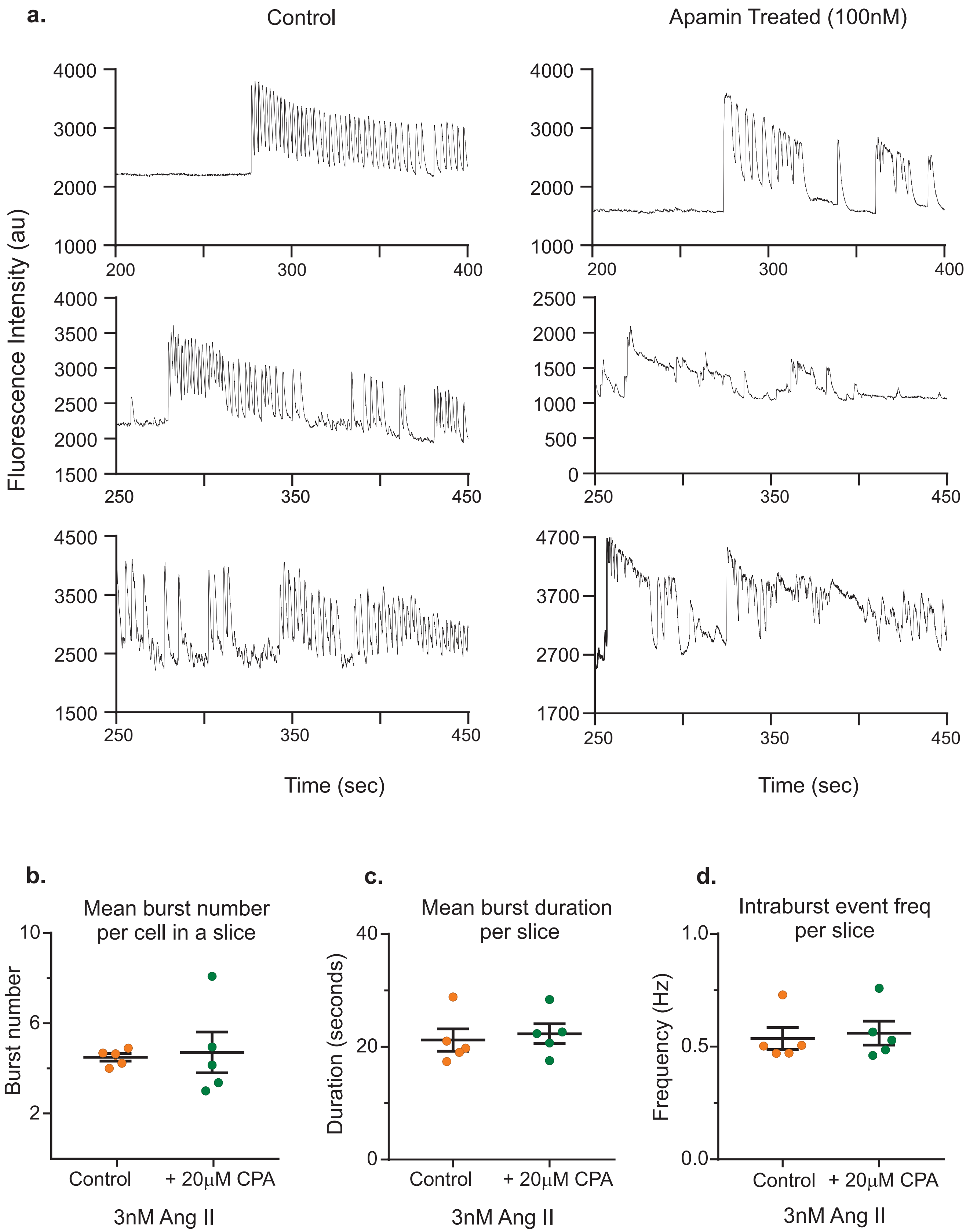
a.
Control
Apamin Treated (100nM)
Fluorescence Intensity (au)
Time (sec)
Time (sec)
b.
Mean burst number per cell in a slice
Burst number
Control
+ 20μM CPA
3nM Ang II
c.
Mean burst duration per slice
Duration (seconds)
Control
+ 20μM CPA
3nM Ang II
d.
Intraburst event freq per slice
Frequency (Hz)
Control
+ 20μM CPA
3nM Ang II

Supplementary Figure 2. Characteristics of calcium response to apamin and CPA. (a) Three examples of control **(left panels)** and 100nM Apamin-treated (right panels) slices. Apamin-treated slices exhibited a fundamentally different calcium response to 3nM Ang II; a great deal of variability was observed from cell to cell/mouse to mouse, with often slower, more infrequent oscillations. **(b-d)** Ca^{2+} from endoplasmic reticular stores does not affect burst characteristics. Slices pre-incubated with 20µM cyclopiazonic acid (CPA) and stimulated with 3nM Ang II did not differ in mean burst number (**b**, means±SEM per adrenal slice(number): control 4.50±0.16, CPA 4.71±0.91, Mann-Whitney test: P = 0.84), duration (**c**, means±SEM per adrenal slice (seconds): control 21.21±2.00, CPA 22.33±1.77, Mann-Whitney test: P = 0.55:), or intraburst frequency (**d**, means±SEM per adrenal slice (Hz): control 0.54±0.05, CPA 0.56±0.05, Mann-Whitney test: P = 0.69. CPA: *n* = 5 slices, 89 cells; No CPA: *n* = 5 slices, 106 cells; mean data from each mouse were represented as a single point in calculating N/experimental condition.

Figure S3

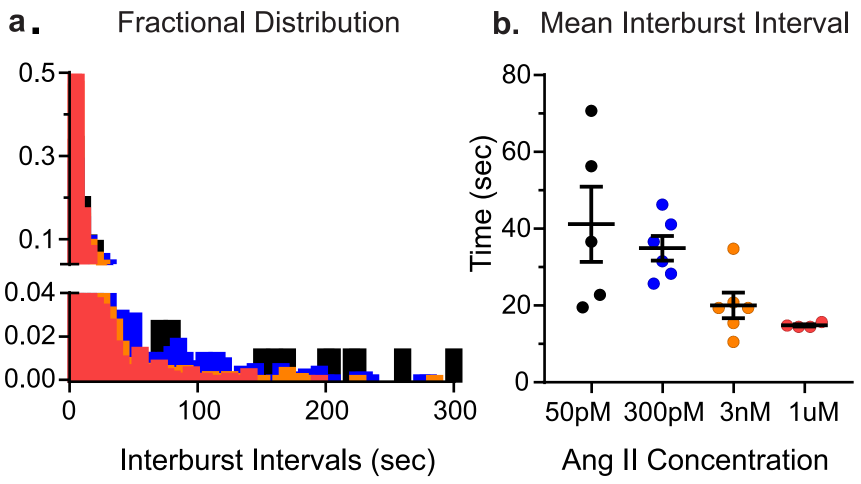

Supplementary Figure 3. The interburst interval is Ang II dose-dependent. **(a)** Fractional distribution of interburst intervals (seconds) and **(b)** mean interburst interval (means±SEM [seconds]: 50pM: 41.2±9.9; 300pM: 34.9±3.2, 3nM: 20.1±3.3, 1µM: 14.9±0.3); Kruskal-Wallis test, P = 0.005; Dunn's multiple comparison test: 50pM vs 1µM (P = 0.027) and 300pM vs 1µM (P = 0.023), non-linear curve fit: $R^2 = 0.46$**,** IC_{50} = 876.49pM. 50pM (*n* = 5, 59 cells), 300pM (*n* = 6, 217 cells), 3nM (*n* = 7, 163 cells) or 1µM (*n* = 4, 95 cells); mean data from each mouse were represented as a single point in calculating N/experimental condition. Bars and symbols are color coded according to experimental condition; 50pM: black, 300pM Ang II: blue, 3nM Ang II: orange, 1µM Ang II: red.

Figure S4

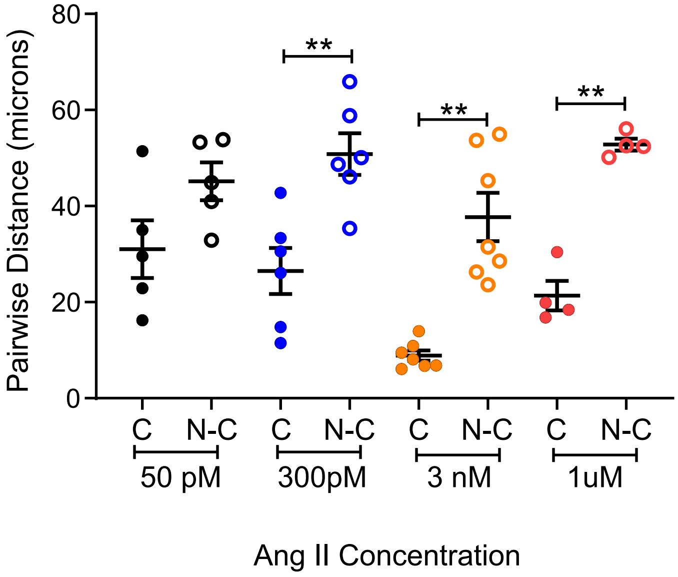

Supplementary Figure 4. zG cell clusters consist of spatially proximal cells at higher concentration of Ang II. Mean pairwise distance is smaller among functionally clustered cells at doses of Ang II 300pM and greater. 1-way ANOVA (P <0.001) with Bonferroni's test; means±SEM (µm): 50pM (n=5): C = 31.04±6.00 vs. N-C = 45.17±3.93, N.S.; 300pM (n=6), C = 26.52±4.79 vs. N-C = 50.83±4.310, P = 0.0006; 3nM (n=7), C = 8.88±1.06 vs. N-C = 37.72±5.03, P = 0.0003; 1µM (n=4), C = 21.39±3.07 vs N-C = 52.82±1.22 P < 0.0001. Symbols are color coded according to experimental condition; 50pM: black, 300pM Ang II: blue, 3nM Ang II: orange, 1µM Ang II: red; open circle: clustered (C) pair, closed circle: non-clustered (N-C) pair.

Figure S5

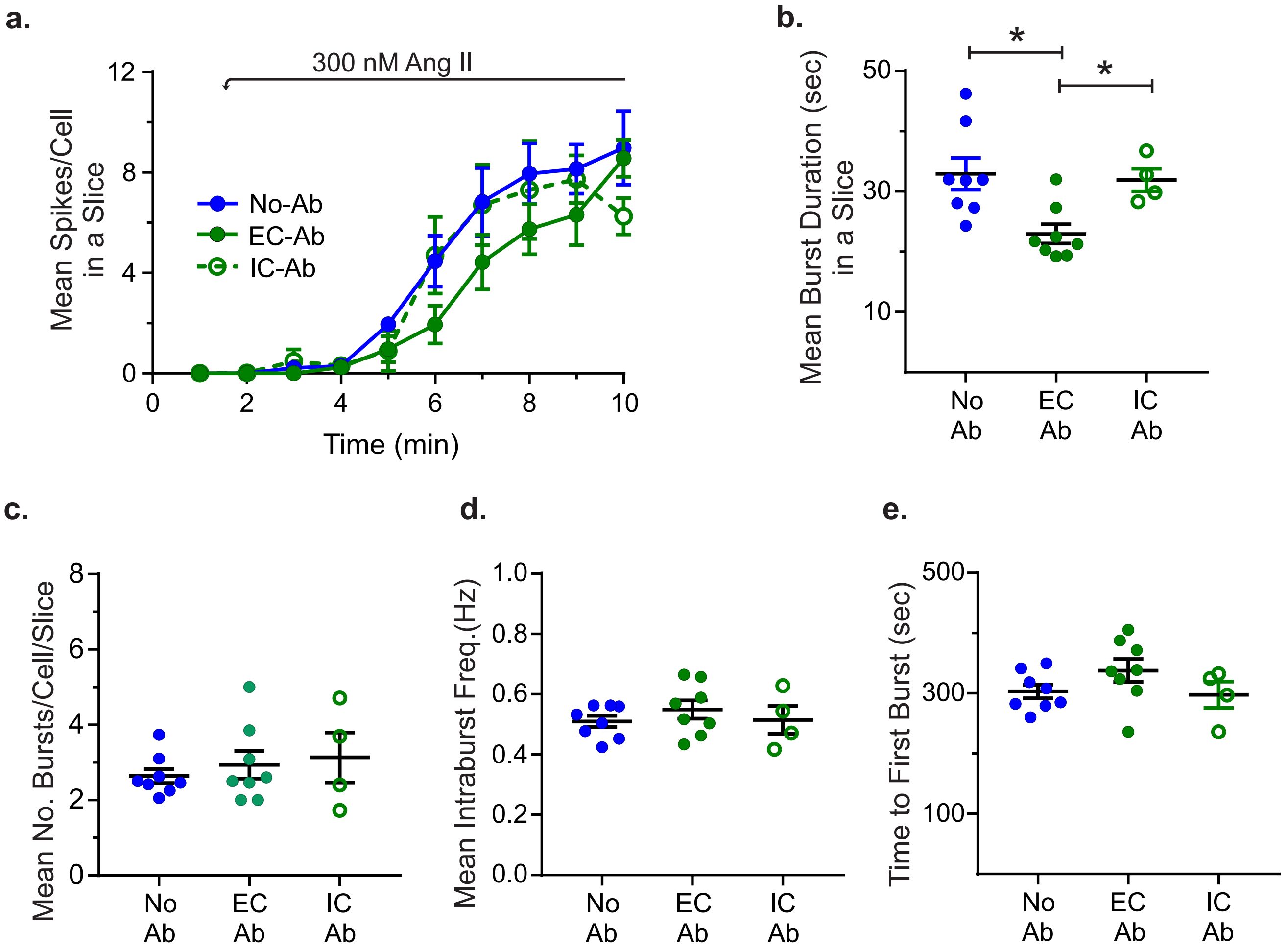
a.
300 nM Ang II
Mean Spikes/Cell in a Slice
No-Ab
EC-Ab
IC-Ab
Time (min)
b.
Mean Burst Duration (sec) in a Slice
*
*
No Ab
EC Ab
IC Ab
c.
Mean No. Bursts/Cell/Slice
d.
Mean Intraburst Freq.(Hz)
e.
Time to First Burst (sec)

Supplementary Figure 5. Disruption of N-cadherin alone had only a modest effect on calcium spike number and burst duration. In one set of experiments, anti-N-cadherin alone (i.e., not with anti-K-cadherin) was used to disrupt cadherin junctions. Slices were incubated in a low calcium (0.3mM) Pipes buffer for 20 minutes, followed by low calcium buffer containing 1mM EGTA with an extracellular N-cadherin antibody (EC-ab, 1:25, n = 8), intracellular Pan-cadherin (IC-ab, 1:25, n = 4), or no antibody (no-ab, vehicle, n= 8) for 40 minutes. The final calcium-replete incubation solution contained no EGTA, 1mM calcium, and incubation antibody (1:25), if applicable (41). **(a-e)** Adrenal slices were stimulated with 300pM Ang II after 1.5 mins of baseline recording. **(a)** Mean spikes per 1 min bin over a 10 min period; EC-Ab treated slices trended toward fewer spikes over time, but 2-way ANOVA showed no significant difference among groups (P = 0.31). **(b)** Mean burst duration/slice was significantly reduced after cell adhesion disruption; means±SE (seconds): no-ab: 32.92±2.6; EC-Ab: 22.97±1.6; IC-Ab: 3.13±1.9; Kruskal-Wallis test: P = 0.0065; Dunn's multiple comparison test: EC-Ab vs IC-Ab *P = 0.047; EC vs no-ab *P = 0.034; IC vs no-ab P > 0.99. Neither, number of bursts/cell/slice (**c,** means±SEM: no-ab: 2.64±0.2; EC-Ab: 2.94±0.4; IC-Ab: 3.13±0.7; Kruskal-Wallis test: P > 0.99), intraburst Frequency (**d**, means±SEM [Hz]: no-ab: 0.510±0.03; EC-Ab: 0.550±0.03; IC-Ab: 0.515±0.05; Kruskal-Wallis test: P = 0.58), nor time to first burst (**e**, means±SEM [sec]: no-ab: 303.1±11.23; EC-Ab: 338.0±18.8; IC-Ab: 297.8±21.8; Kruskal-Wallis test: P = 0.25) differed from control experiments. **(a-e)** Mean data from each mouse were represented as a single point in calculating N/experimental condition. Symbols are color coded according to experimental condition; No-Ab: blue solid, EC-Ab: green solid, IC-Ab green open.

Figure S6

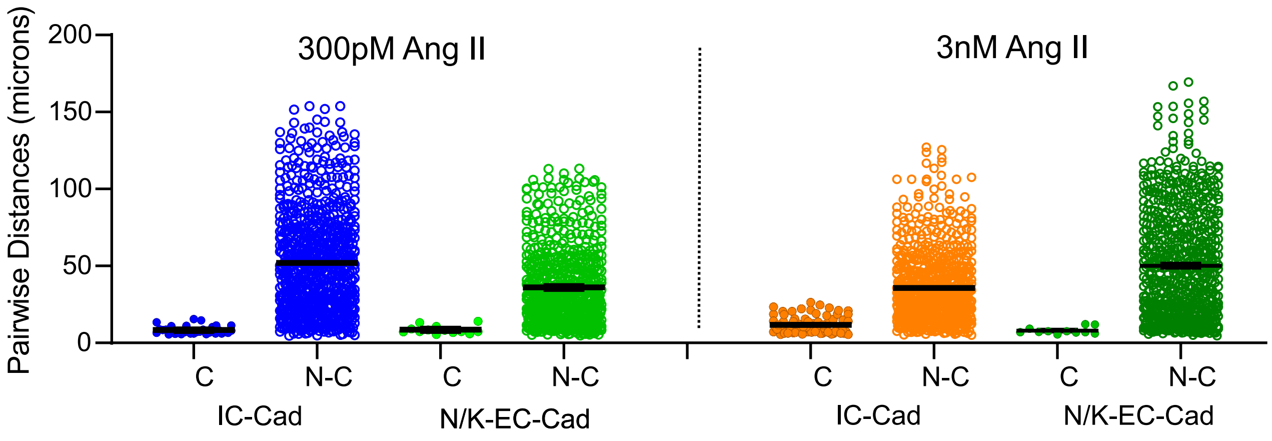

Supplementary Figure 6. Pairwise distances of clustered vs. non-clustered zG cell pairs collapsed across cadherin disruption experiments. zG cell pairs are categorized by functional cluster analysis as clustered (C) or non-clustered (N-C). Regardless of disruption protocol, clustered pairs are located proximal to each other, while non-clustered cells are widely distributed across the slice. Means±SEM (µm): 300pM IC-Ab (n=6 experiments, 812 pairs), C = 8.38±0.47, N-C = 51.94±1.22; 300pM EC-Abs (n= 6 experiments, 685pairs): C = 8.56±0.73 N-C = 36.06±0.94; 3nM IC-Ab (n=6 experiments, 848 pairs), C = 11.72±0.64, N-C = 35.81±0.82; 3nM EC-Abs (n=6 experiments, 732 pairs), C = 8.00±0.67, N-C = 50.19±1.34. Symbols are color coded according to experimental condition; IC-Ab + 300pM Ang II: blue, EC-Abs + 300pM Ang II: light green, IC-Ab + 3nM Ang II: orange, EC-Abs + 3nM Ang II: dark green.
