# Supplementary material for: Angiotensin II induces coordinated calcium bursts in aldosterone-producing adrenal rosettes
Source: Nat Commun. 2020 Apr 3;11:1679. doi: 10.1038/s41467-020-15408-4 (PMC7125102; doi:10.1038/s41467-020-15408-4)
Supplement: Supplementary file 3 — Description of Additional Supplementary Files [file 41467_2020_15408_MOESM3_ESM.pdf]

Description of Additional Supplementary Files

File Name: Supplementary Movie 1

Description: Ang II evokes robust calcium oscillations in zG cells expressing GCaMP3. Images were captured at 20 Hz and compiled showing basal and 3nM Ang II-evoked activity (as indicated). Video was resampled at ~5x real-time.
