# Supplementary material for: Angiotensin II induces coordinated calcium bursts in aldosterone-producing adrenal rosettes
Source: Nat Commun. 2020 Apr 3;11:1679. doi: 10.1038/s41467-020-15408-4 (PMC7125102; doi:10.1038/s41467-020-15408-4)
Supplement: Supplementary file 5 — Reporting Summary [file 41467_2020_15408_MOESM5_ESM.pdf]

nature research

Corresponding author(s): Mark Beenhakker

Last updated by author(s): Jul 9, 2019

Reporting Summary

Nature Research wishes to improve the reproducibility of the work that we publish. This form provides structure for consistency and transparency in reporting. For further information on Nature Research policies, see Authors & Referees and the Editorial Policy Checklist.

Statistics

For all statistical analyses, confirm that the following items are present in the figure legend, table legend, main text, or Methods section.

n/a	Confirmed	
☐	☒	The exact sample size (*n*) for each experimental group/condition, given as a discrete number and unit of measurement
☐	☒	A statement on whether measurements were taken from distinct samples or whether the same sample was measured repeatedly
☐	☒	The statistical test(s) used AND whether they are one- or two-sided<br>*Only common tests should be described solely by name; describe more complex techniques in the Methods section.*
☐	☒	A description of all covariates tested
☐	☒	A description of any assumptions or corrections, such as tests of normality and adjustment for multiple comparisons
☐	☒	A full description of the statistical parameters including central tendency (e.g. means) or other basic estimates (e.g. regression coefficient) AND variation (e.g. standard deviation) or associated estimates of uncertainty (e.g. confidence intervals)
☐	☒	For null hypothesis testing, the test statistic (e.g. *F*, *t*, *r*) with confidence intervals, effect sizes, degrees of freedom and *P* value noted<br>*Give P values as exact values whenever suitable.*
☒	☐	For Bayesian analysis, information on the choice of priors and Markov chain Monte Carlo settings
☒	☐	For hierarchical and complex designs, identification of the appropriate level for tests and full reporting of outcomes
☐	☒	Estimates of effect sizes (e.g. Cohen's *d*, Pearson's *r*), indicating how they were calculated

Our web collection on statistics for biologists contains articles on many of the points above.

Software and code

Policy information about availability of computer code

Data collection	Haimatsu's HCImageLive, 3i's Slidebook 6.0
Data analysis	Microsoft Excel, ImageJ, Graphpad Prism 7, Caltracer3beta from the Yuste Laboratory (blogs.cuit.columbia.edu/rmy5/files/2018/02/caltracer3beta.zip), Custom Matlab scripts

For manuscripts utilizing custom algorithms or software that are central to the research but not yet described in published literature, software must be made available to editors/reviewers. We strongly encourage code deposition in a community repository (e.g. GitHub). See the Nature Research guidelines for submitting code & software for further information.

Data

Policy information about availability of data

All manuscripts must include a data availability statement. This statement should provide the following information, where applicable:

- Accession codes, unique identifiers, or web links for publicly available datasets
- A list of figures that have associated raw data
- A description of any restrictions on data availability

All datasets generated during and/or analyzed during the current study are available from the corresponding author on reasonable request.

Field-specific reporting

Please select the one below that is the best fit for your research. If you are not sure, read the appropriate sections before making your selection.

☒ Life sciences ☐ Behavioural & social sciences ☐ Ecological, evolutionary & environmental sciences

For a reference copy of the document with all sections, see nature.com/documents/nr-reporting-summary-flat.pdf

Life sciences study design

All studies must disclose on these points even when the disclosure is negative.

Sample size	When possible, we used a sample size estimated a priori to provide sufficient power (0.8) using G*Power 3.1.9.2 software with a moderate effect size. However, due to limitations of colony size, some experiments were done with fewer animals than suggested by power analysis.
Data exclusions	A mouse was excluded if adrenal slices did not respond to 3nM Angiotensin II, indicating poor GCaMP expression or poor tissue health.
Replication	Experimental conditions were repeated across multiple mice. When conducting statistical analysis, each mouse contributed 1 data point per condition (n=1), regardless of the number of slices/cells analyzed from that mouse.
Randomization	We used young adult male GCaMP3/cre expressing mice (40-100 days old) in the order in which they were available. For imaging studies, adrenals from only one mouse were sectioned on a given day, and each slice for a given experimental condition was randomly selected from a pool of intact, macroscopically similar sections. For aldosterone secretion studies, sections were divided randomly into wells, and well randomly assigned experimental condition, with 2-4 sections per well depending on the experiment.
Blinding	Data acquisition was not performed blind; having the investigator unaware of the solution content while imaging slices was not practical. Selection of ROIs in the zG layer was done without knowledge of experimental condition. Event detection was automated using a Matlab script followed by validation by an investigator who corrected detection errors. Aldosterone measurements were automated using an automatic gamma counter

Reporting for specific materials, systems and methods

We require information from authors about some types of materials, experimental systems and methods used in many studies. Here, indicate whether each material, system or method listed is relevant to your study. If you are not sure if a list item applies to your research, read the appropriate section before selecting a response.

Materials & experimental systems

n/a	Involved in the study	
☐	☒	Antibodies
☒	☐	Eukaryotic cell lines
☒	☐	Palaeontology
☐	☒	Animals and other organisms
☒	☐	Human research participants
☒	☐	Clinical data

Methods

n/a	Involved in the study	
☒	☐	ChIP-seq
☒	☐	Flow cytometry
☒	☐	MRI-based neuroimaging

Antibodies

Antibodies used	Primary antibodies: anti-N-cadherin (Novus Biologicals, NBP2-38856, immunohistochemistry); anti-N-cadherin (Sigma Aldrich, C3865, calcium switch assay), anti-K-cadherin (ThermoFisher Scientific, MA1-06305, calcium switch assay), Anti-K-cadherin (Abcam, ab133632, immunohistochemistry), anti-Pan-cadherin (Sigma Aldrich, C1821, calcium switch assay); Secondary Antibody: Alexa Fluor 488-conjugated goat anti rabbit IgG (Invitrogen, A-11034)
Validation	All antibodies were validated by the manufacturer.

Animals and other organisms

Policy information about studies involving animals; ARRIVE guidelines recommended for reporting animal research

Laboratory animals	These studies used genetically modified male mice on a mixed C57Bl6/SV129 background; ages ranged from 40-100 days old.
Wild animals	The Study did not involve wild animals.
Field-collected samples	The Study did not involve field-collected samples.
Ethics oversight	All experiments were performed in accordance with the National Institutes of Health Guide for the Care and Use of Laboratory Animals and approved by the University of Virginia Animal Care and Use Committee.

Note that full information on the approval of the study protocol must also be provided in the manuscript.
